# The future of pharmaceutical care in France: a survey of final-year pharmacy students' opinions

**DOI:** 10.1186/1472-6904-11-6

**Published:** 2011-05-25

**Authors:** Clémence Perraudin, Françoise Brion, Olivier Bourdon, Nathalie Pelletier-Fleury

**Affiliations:** 1 Faculté de Médecine Paris-Sud Paris XI, Le Kremlin-Bicêtre, France; 2 Institut National de la Santé et de la Recherche Médicale (INSERM) Unité 988, Villejuif, France; 3 Centre National de la Recherche Scientifique (CNRS) UMR 8211, Villejuif, France; 4 Ecole des Hautes Etudes en Sciences Sociales (EHESS), Paris, France; 5 Faculté de Pharmacie, Université Paris Descartes, France; 6 APHP Hôpital Robert Debré, Paris, France

## Abstract

**Background:**

In the last decades, the provision of pharmaceutical care by community pharmacists has developed in OECD countries. These developments involved significant changes in professional practices and organization of primary care. In France, they have recently been encouraged by a new legal framework and favored by an increasing demand for health care (increase in the number of patients with chronic diseases) and reductions in services being offered (reduction in the number of general practitioners and huge regional disparities).

Objectives: This study aimed to investigate final-year pharmacy students' opinions on 1/expanding the scope of pharmacists' practices and 2/the potential barriers for the implementation of pharmaceutical care. We discussed these in the light of the experiences of pharmacists in Quebec, and other countries in Europe (United Kingdom and the Netherlands).

**Methods:**

All final-year students in pharmaceutical studies, preparing to become community pharmacists, at the University Paris-Descartes in Paris during 2010 (n = 146) were recruited. All of them were interviewed by means of a questionnaire describing nine "professional" practices by pharmacists, arranged in four dimensions: (1) screening and chronic disease management, (2) medication surveillance, (3) pharmacy-prescribed medication and (4) participation in health care networks. Respondents were asked (1) how positively they view the extension of their current practices, using a 5 point Likert scale and (2) their perception of potential professional, technical, organizational and/or financial obstacles to developing these practices.

**Results:**

143 (97.9%) students completed the questionnaire. Most of practices studied received a greater than 80% approval rating, although only a third of respondents were in favor of the sales of over-the-counter (OTC) drugs. The most significant perceived barriers were working time, remuneration and organizational problems, specifically the need to create a physical location for consultations to respect patients' privacy within a pharmacy.

**Conclusions:**

Despite remaining barriers to cross, this study showed that future French pharmacists were keen to develop their role in patient care, beyond the traditional role of dispensing. However, the willingness of doctors and patients to consent should be investigated and also rigorous studies to support or refute the positive impact of pharmaceutical care on the quality of care should be carried out.

## Background

Faced with increasing demand for health care and reductions in services being offered, particularly by general practitioners (GPs), questions are increasingly being raised in various regions of France about the health care principles of proximity, availability and access. To alleviate these problems, a health-care reform law was adopted in 2009 (known as the *"Hôpital, Patients, Santé, Territoires" *or "HPST" law). Primary care is at the forefront of this reform, and for the first time, a legislative framework is broadening the role of pharmacists in providing these services. After being merely a dispenser of medications (article R4235-48 of the Public Health law), the pharmacist is now being assigned responsibilities in front-line health care, health-care coordination, screening and therapeutic education (article 38 of the HPST law).

In 2009, there were 22,386 pharmacies in metropolitan France and 53,460 practicing pharmacists, with an average of one pharmacy for every 2,849 inhabitants [1]. The geographical distribution of pharmacies in France is relatively homogeneous because the licensing of pharmacies is regulated by demographic criteria, with one pharmacy per 2,500 or 3,000 inhabitants according to the size of the locality (law of September 11, 1941). With these figures, France has one of the highest pharmacy densities in Europe. The over-the-counter (OTC) market in France is different from neighboring European countries. Drugs provided without prescription account for only 17% of the volume of items sold in pharmacies [2].

The challenge today is to understand the conditions and consequences of a number of technical, organizational and social innovations. No scientific study has examined this issue with respect to the pharmacists' professional practices in France. One could hypothesize that the pharmacist model, in the meaning used by Hepler and Strand [3] as a dispenser of pharmaceutical care, may become the practice in France as well. In any case, the current discrepancies between the demand for and supply of health care provide a context that is conducive to this model, and the legal framework encourages this trend. However, the opposite hypothesis may also be valid; the redefinition of the pharmacist's profession [4] and professional, economic and/or organizational boundaries together with normal resistance to change may become obstacles to the implementation of this reform [5].

The objective of the present study was to analyze the opinions of final-year pharmacy students on expanding the scope of pharmacists' practices in France and thereby assess the potential barriers to adopting new practices.

## Methods

### Survey population

The survey population consisted of all final-year students in pharmaceutical studies who were preparing to become community pharmacists at the University of Paris-Descartes in Paris during 2010 (n = 146). All of these students were questioned at the end of their 6-month, final-year practical experience of working in a community pharmacy. We took advantage of the fact that these students were required to return to the university between June 21 and 28 for an oral examination to validate their practical experience. During this week, after we obtained their oral consent, we administered a printed questionnaire to all the students. These questionnaires were collected at the end of the examination.

### Questionnaire

In France, the pharmacist's mission already extends beyond merely the sale of medications. They also provide advice to patients and sell OTC drugs without medical prescriptions. However, their mission is far from providing patient-centered services [6]. Drawing on the HSPT law and experience in other countries [7-9], we developed a questionnaire describing nine practices of pharmacists. These are summarized in Table 1 and are arranged in four dimensions: (1) screening and chronic disease management, (2) medication surveillance, (3) pharmacy-prescribed medication and (4) participation in health-care networks.

**Table 1:** Description of the nine pharmacists' practices.

Dimension 1: Screening and chronic disease management	
**The pharmaceutical consultation**	Individual interview in a confidential area to inform and counsel the patient by explaining the treatment, its side effects, and drug interactions, and any follow-up to be adopted.

**Therapeutic education**	Practical tools for the patient to acquire skills to manage their disease and its care and supervision in partnership with health-care providers.

**The pharmacist as a coordinator of care**	A protocol allowing the community pharmacist, chosen by the patient, to periodically renew chronic treatments, adjust dosage (if necessary), and make medication-use reviews (side effects, observance, follow-up) at a doctor's request or with his consent.

**Screening**	Offering screening procedures for certain ailments to patients using easily administered tests such as blood pressure, expiratory flow rate, and blood-sugar levels.

**Dimension 2: Medication surveillance**	

**The electronic pharmaceutical record (e-pr)**	Making an electronic file for each patient containing all drugs dispensed to the patient during the last four months for his or her own personal consumption, with or without medical prescription, in any pharmacy that is equipped for such recording.

**The pharmaceutical opinion**	A professional opinion, under the pharmacist's authority, on the pharmaceutical appropriateness of one or a series of treatments to be dispensed by the pharmacist. This is to be communicated on a standardized form to the prescriber of the medication and/or to the patient when the pharmacist recommends a revision or to justify his refusal to dispense a medication as prescribed.

**Dimension 3: Pharmacy-prescribed medication**	

**Prescription for minor ailments**	Dispensing certain medications without a medical prescription or advising patients to consult a doctor, following appropriate questioning of the patient to determine the gravity of the symptoms of his or her ailment.

**Sales of over-the-counter drugs**	Direct public access to medications referred to as "pharmaceutical products" in specific, clearly identified locations in very close proximity to where medications are dispensed, and providing the public with information from respected health-care authorities relative to the appropriate use of these products.

**Dimension 4: Participation in health-care networks**	

**Pharmacotherapeutic consultation groups**	Group discussions with GPs and/or other specialists to discuss the clinical situation of patients for whom they jointly provide care, such as in the framework of health-care networks.

The respondents were asked two questions: (1) how positively they view the extension of their current practices (using a 5-point Likert scale from "strongly favorable" to "strongly unfavorable") and (2) their perception of potential professional, technical, organizational and/or financial obstacles to developing these practices. For this latter variable, several response categories were provided. Upon completion of the questionnaire, we collected data on the respondents' characteristics, such as age, gender, motivation for selecting the community-pharmacy option in their studies (four alternatives) and the degree to which their practical experience working in a community pharmacy reflected their expectations (using a Likert scale with five choices from "completely" to "not at all").

## Results

The questionnaire was completed by 143 of the 146 the students (97.9%) enlisted. The three remaining students left before the examination was completed and did not complete the questionnaire.

### Sample characteristics

The majority of the respondents were women (73.9%), and the average age was 25.1 ± 1.6 years. These students chose the community-pharmacy option in their studies because of their desire for patient contact (46.1%), their wish to create an enterprise (11.2%) or a combination of these reasons (28.7%). Only 8.4% chose the community-pharmacy option by default, and 5.6% did not know how to answer this question. For the majority (79.2%), their experience working in a community pharmacy met their expectations "completely" or "almost completely," but 17.1% indicated "averagely" and 3.5% answered "little" or "not at all."

### Opinions on expanding the scope of pharmacists' practices

**Figure 1:**
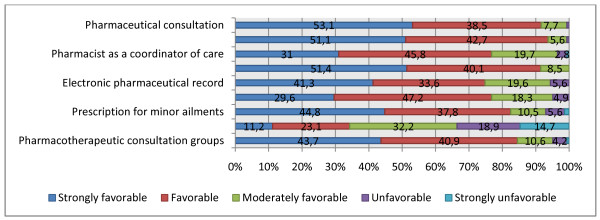
**Final-year pharmacy students' opinions on expanding the scope of the pharmacist's practices (%)**.

Figure 1 shows the respondents' opinions on expanding the scope of the pharmacist's practices. Eight of the 9 practices studied received a greater than 80.0% approval rating ("favorable" or "strongly favorable"). However, only 34.3% had "favorable" or "strongly favorable" opinions about expanding the sales of OTC drugs. We should note that 14.7% felt "Strongly unfavorable" about this practice.

### Obstacles to developing new practices

**Figure 2:**
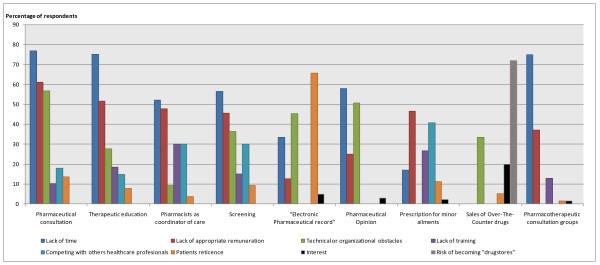
**The potential obstacles to developing new pharmacists' practices (%)**.

Figure 2 summarizes the potential obstacles to developing new pharmacists' practices. Lack of time and appropriate remuneration constitute the major barriers to developing the four practices under the "screening and chronic disease management" heading. These items were respectively checked by 77.0% and 61.2% of respondents with respect to "pharmaceutical consultation" and by 75.2% and 51.8% for developing "therapeutic education." Also, organizational obstacles, specifically the need to create a physical location for consultations to respect patients' privacy within a pharmacy, were seen by most respondents (56.8%) as a brake on "pharmaceutical consultation" and by more than a third (36.4%) as limiting the development of "screening." Finally, lack of training and competition with doctors were considered to be significant impediments to developing "pharmacists as coordinators of care," with 30.0% of the respondents checking these two items. Competition with doctors was cited by 30.0% as an obstacle for "screening."

With respect to the "medication surveillance" dimension, half of the respondents checked technical obstacles, specifically those involving computer hardware and the problem of sharing information between health-care professionals. These were cited by 45.5% as factors limiting "electronic pharmaceutical record" (e-pr) development and by 50.7% as factors limiting "pharmaceutical opinion." Time was also mentioned as a factor constraining e-pr development by a third of the respondents (33.6%), and by more than 50.0% (57.9%) as limiting "pharmaceutical opinion," whereas remuneration was infrequently selected as a limiting factor. It is particularly striking that nearly two-thirds of the respondents (65.7%) considered patient reticence as a potential obstacle to e-pr development.

As for the "pharmacy-prescribed medication" dimension for minor ailments, in addition to the lack of remuneration, which was considered by nearly half (46.5%) of the respondents as a barrier, we also found problems with competing with doctors (40.8%) and, to a lesser extent, lack of training (26.8%). Less than one-fifth of the respondents (16.9%) mentioned lack of time for this dimension. For "selling OTC medications," the notable finding was the high percentage (72.1%) who perceived the risk of pharmacies becoming "drugstores" as an obstacle to its development. Added to this were organizational problems, specifically the space available for these items in pharmacies (33.6% of respondents). Finally, for "participation in health-care networks," time and (to a lesser degree) remuneration were seen as the principal obstacles (75.0% and 37.1%, respectively).

## Discussion

### Pharmaceutical care

The concept of pharmaceutical care originated in an article by Hepler and Strand [3]. Although there are several definitions of this concept, most agree that a pharmacist's commitment to the patient is to attain the patient's health objectives. This concept has become widely used in North America, particularly in Quebec, but it has received less recognition in Europe [10], with the exception of the United Kingdom and the Netherlands [8]. The pharmacists' practices considered in the present study are integrated into the concept of pharmaceutical care that is defined by the *Ordre des Pharmaciens du Québec *(The College of Pharmacists in Quebec) as: "all the acts and services that the pharmacist is required to provide to patients, in order to improve the quality of their lives by attaining the pharmacotherapeutic objectives of prevention, cure or the provision of palliative care." [11]

### Screening and chronic disease management

In Quebec, community pharmacists can, during a private pharmaceutical consultation with a patient, provide the following: develop a pharmaceutical care plan and the objectives to be attained; write a pharmaceutical opinion with suggestions to the patient's doctor to revise the patient's treatment; conduct simple tests in follow-up treatment, such as blood pressure; take the time to explain the treatment and the ailments; and listen to what the patient has to say [12,13]. The pharmacist can also participate in therapeutic education projects by showing patients how to modify their lifestyle to reduce their symptoms and improve their short-, medium- and long-term health status [14]. New approaches that give pharmacists the right to write out prescriptions are emerging elsewhere, such as in the United States and the United Kingdom, through establishing a protocol between the doctor, the pharmacist and the patient, defining the procedures and responsibilities of each person and developing the health-care project to be adopted. Collaborative drug-therapy management is practiced throughout the United States and is officially recognized in 25 states and by the federal government (Armed Forces and Veterans Affairs) [15]. In the United Kingdom, following a public consultation and under advice from the Committee on Safety of Medicines and the Medicines Commission, Ministers in 2003 agreed to the implementation of supplementary prescribing [9]. The procedures can be followed to different degrees, such as ranging from the initiation of treatment through to modifying and/or renewing the medication therapy.

In France, the pharmacist's involvement in providing care for patients with chronic ailments is one of the pillars of this recently adopted legislation. Due to health-care professionals' initiatives (pharmacists, doctors and nurses), co-operation agreements (defined since July 21, 2009 by Article 4011-1 of the Public Health law) may be signed, which follow the consultation with the *Haute Autorité de Santé *(French National Authority for Health) and authorization by the *Agence Régionale de Santé (Regional Health Agency)*. The objective is to transfer activities and/or care and to reorganize intervention procedures with patients between the different health-care professionals according to their respective knowledge and experience levels. In our study, the great majority of young pharmacists look favorably on implementing these measures. Nonetheless, according to our respondents, lack of time and remuneration constitute barriers for developing these new practices. In France today, pharmacists are essentially remunerated through profit margins on the medications they sell; therefore, the present remuneration system would have to be modified, and several alternatives can be envisaged. In Quebec, pharmacists are moving towards a system of being paid per pharmaceutical act [16]. In 2009, pharmacist representatives in France proposed (in the Rapport Rioli [17]) a mixed system of remuneration, which would make distinctions between the profit margins on medications sold, the fees for pharmaceutical acts and reimbursements for services provided. This proposal was inspired by the 2005 agreement signed between the National Health Service (NHS) and pharmacists [18] in the United Kingdom. This agreement identifies three levels of intervention, which differ according to the services offered, the source of funding and the agreements signed with the government. The *Rapport Rioli *also considered the roles that the compulsory health insurance system and complementary health plans should play in providing these services. This agreement was signed by eight pharmacist associations and was approved by the Ministry of Health. There are already a few initiatives wherein pharmacists are remunerated independently of medication sales. The insurance company GMF-Allianz has developed a model contract that remunerates pharmacists for their pharmaceutical consultations [19]. *La Mutuelle de la Région Lyonnaise *(complementary health plan of the Region of Lyon) is proposing a personalized prevention assessment for pharmacists. This assessment is made when a new contract is signed, it is renewed every two years, and each act is reimbursed up to a maximum of 22€ per beneficiary. Thus, a debate regarding a new system of remuneration for pharmacists is taking place in France.

In our study, the lack of appropriate training was perceived to represent a significant obstacle to developing chronic disease management, particularly in the context of increasing the coordinating role of pharmacists. In 2006, the World Health Organization and the International Pharmaceutical Federation (FIP) called for a new paradigm in pharmaceutical care that would require pharmacists to go beyond their training as experts in chemistry and their knowledge of pharmaceutical products. This paradigm called on pharmacists to understand and apply the principles involved in managing medical therapies, such as the clinical and social roles of pharmacies, communication, health promotion and ethics [20,21]. The FIP also recommended increasing vocational training for pharmacists [22]. In the United Kingdom, for example, pharmacists can only provide supplementary prescriptions after they train at a higher education institution and complete a "period of learning in practice" in accordance with the Royal Pharmaceutical Society of Great Britain curriculum. Currently, the practice of clinical pharmacy is poorly developed in France, in both hospitals and in the community. Starting in the fall of 2010, a common first year may be established for all students in medicine, pharmacy, dentistry and midwifery, and this may become an opportunity to create a common basis for these future health-care professionals and encourage tomorrow's collaborative practices.

Pharmacies are providing more screening practices in France, which are resulting from the combined initiatives of pharmacies collaborating with each other [23] or during awareness campaigns such as "Diabetes Day." In light of what has been happening in Germany, pharmacies could offer their patients the possibility of monitoring their blood pressure, blood-sugar levels, lipid values, body mass index or waistline [24]. In addition to the problems of remuneration and available working-time, the principal obstacles mentioned by our respondents concerning these practices were pharmacy layout, lack of space for meeting patients confidentially and competition with GPs, which is discussed below (see Pharmacy-prescribed medication). Designated spaces in pharmacies for respecting privacy or separate rooms for these consultations have to be created to develop this screening activity and to provide care for chronic ailments. The *Ordre des Pharmaciens *(The College of Pharmacists in France) mentioned this need in July 2006 [25]. Rapport et al. (2009) [26] were concerned with the impact of the community pharmacy setting on professional practice and sense of self. They emphasized the importance of separate enclosed consultation rooms in pharmacies in the United Kingdom. Providing such space would increase the degree of confidentiality during consultations and heighten the professional's sense of being a valuable consultant and diagnostician. However, typically, these spaces are not clearly reserved for consultations, and they are often used for stocking products due to the lack of space in small pharmacies. Rapport *et al. *concluded that, unlike in the case of GPs, a consultation room in a pharmacy tends to be a place where the patient is encouraged to buy, be served and move on instead of a place where the patient can expect to spend time with a health-care professional.

### Medication surveillance

For many years, strategies for using electronic health records (e-hr) have been very different from one country to another. One of the first uses of e-hr was for medical prescriptions [27]. In France, an initial e-hr project *Dossier Médical Personnalisé *was adopted into law on August 13, 2004. It was abandoned due to problems with its application, such as deadlines not being respected and divergent interpretations of the limits and uses of the e-hr. The purpose was to create an e-hr for each person in France, which would include all of his or her past and present medical information. The e-pr developed independently of the e-hr. The e-pr is a pharmacist's professional tool designed to ensure the safe dispensing of medications. It records all medications delivered to a patient during the last four months from every pharmacy, which are linked into the system. By consulting the e-pr, the pharmacist can identify medicinal interactions and treatment redundancies. The National Consultative Committee of the *Ordre des Pharmaciens*, which initiated the e-pr, has been mandated by law (n° 2007-127 of January 30, 2007) to use this system. In the future, this will facilitate the implementation of the e-hr program. In our study, the great majority of the young pharmacists were open to creating these systems. In 2010, nearly 10 million e-prs had already been created in 18,000 pharmacies. However, obstacles to this initiative have been identified, and patient reticence is the most important. The patient has the right to refuse the creation of their e-pr. This is consistent with results of a recent study [28], which gathered the opinions of patients, GPs and community pharmacists on the development of an electronic transfer system for prescription-related information between GPs and community pharmacies. All groups acknowledged the potential benefits of a full, primary-care information system, but GPs and patients had reservations about allowing community pharmacists to access parts of their medical records that did not concern medications. Technical problems (such as slow internet connections and lack of standardized materials and software) and time were also cited as major obstacles to developing the e-pr. Our respondents also referred to other obstacles, such as patients opting out of the e-pr and deciding on what data to include in it. These obstacles and the technical issues contribute greatly to the time constraints on e-pr development. This would explain why, unlike with other practices, the time constraint was not accompanied by the issue of remuneration.

Pharmaceutical opinion is a procedure from Quebec. In 1984, the *Ordre des Pharmaciens du Québec *defined this as "a pharmacist's judgment on the value of a medication or medicinal treatment following his or her analysis of the patient's e-pr " [29]. Therefore, it is based on the development of an e-pr. Since 1978, pharmacists have been reimbursed for this development by the *Régie de l'Assurance-Maladie du Québec *(Quebec provincial drug plan) [30]. In France, pharmacists often contact prescribing doctors to revise a prescription (regarding dosage, appropriateness, or errors), but there are no formal procedures for transmitting such information. These are considered telephone contacts and are not billed by the pharmacist. This concept of pharmaceutical opinion is nevertheless the subject of a whole chapter in the French practical training guide for final-year pharmacy students [31]. Moreover, in our study, we found that respondents supported expanding this procedure but reported that the practice of pharmaceutical opinion faced major obstacles in terms of the time this would require (independently of remuneration) and the technical problems. Kröger et al had already discussed these obstacles in their 2000 study that aimed to identify factors that influenced Quebec community pharmacists to bill for a pharmaceutical opinion or for a refusal to dispense. The typical pharmacist who billed for opinions or refusals in Quebec was < 45 years of age, had attended a continuing education program on this topic and believed that billing for interventions was important. He or she handled a mean daily volume of 100-250 prescriptions, used a decision-support computer program and had sufficient technical staff assistance [32].

### Pharmacist-prescribed medication

One important finding in our research was that our respondents wanted to increase their status as pharmacist-prescribers for minor ailments (82.6%) but not to increase their sales of OTC drugs (34.3%). This reflects the recent debate in France over selling medications in supermarkets [33]. The majority of tomorrow's pharmacists think that pharmacies are not drugstores. This is consistent with the position of the professional association in defending its monopoly. France is one of the last European countries along with Spain, Italy and Greece where pharmacies have a monopoly over all medications (the sale and distribution of all medications whether or not they require a prescription) [34]. In our study, the problem of competing with doctors for pharmacy-prescribed medications echoes the debate over the recognition of pharmacists prescribing for minor ailments. Thus, even if the official representatives of the profession are ready to implement this, a survey reported in 2005 in the *Quotidien du médecin *showed that 6 out of 10 GPs were still opposed to pharmacists prescribing for low-risk ailments [35]. Since 2005, pharmacists have been authorized to dispense medications for contraception and for breaking tobacco addiction.

### Participation in health-care networks

The practice of direct collaborations between pharmacists and other health-care professionals (particularly GPs) to discuss a patient's clinical case has been borrowed from the "pharmacotherapeutic consultation groups" (FTOs) in the Netherlands [8], where these collaborations began in the 1990s to improve co-ordination between health professionals. In France, such practices appear to occur infrequently, but they are, nevertheless, developing through the extension of health-care networks and the increased participation of pharmacists in these networks [36]. Time and remuneration, according to our respondents, were the principal obstacles to developing this practice.

### Limitations

This study has several limitations. First, the respondents were young Parisian pharmacy students, and their opinions may not reflect those of pharmacists throughout France, given the urban setting of their practices; however, even if Paris is not one of the areas most exposed to problems of proximity and access to health care, the respondents were in favor of (or strongly in favor of) to developing the new practices included in our study. Second, the results of this opinion survey would have been very different, particularly with respect to the obstacles to developing new practices, if the respondents had already been established pharmacists. Our choice of final-year pharmacy students, in addition to the fact that they were accessible, was justified because all respondents would soon be affected by the current reform. Third, this is a study of opinions from the pharmacist's perspective, and the results cannot be seen as predictors of the adoption and application of the reform. They have to be considered alongside the opinions of the two other major protagonists: doctors and patients. This merits subsequent research.

## Conclusions

Overall, the final-year pharmacy students participating in this study, who are tomorrow's pharmacists, held favorable opinions toward developing new practices that are more focused on the patient. However, they saw many obstacles for themselves in the diffusion of these practices. The most significant obstacles were remuneration, working time and organizational and technical problems. The *Ordre des Pharmaciens *has proposed solutions to these problems based on the experiences of other countries. However, nothing can be achieved without creating cooperation contracts with other health-care professionals, particularly doctors, to provide care for chronic ailments. The patient's consent must also be obtained. The opinions of doctors and patients remain as open questions.

## Competing interests

The authors declare that they have no competing interests.

## Authors' contributions

CP led the design of the study, collected the data, performed the statistical analysis and wrote the manuscript. FB and OB contributed to the revising of the questionnaire and the manuscript and acquisition of data. NPF led the design of the study and wrote the manuscript. All authors read and approved the final manuscript.

## Pre-publication history

The pre-publication history for this paper can be accessed here:

http://www.biomedcentral.com/1472-6904/11/6/prepub
